# Feasibility, Acceptability and Limitations of Speech and Language Telerehabilitation during COVID-19 Lockdown: A Qualitative Research Study on Clinicians’ Perspectives

**DOI:** 10.3390/healthcare9111503

**Published:** 2021-11-05

**Authors:** Luisa Cacciante, Błażej Cieślik, Sebastian Rutkowski, Anna Rutkowska, Katarzyna Kacperak, Tomasz Kuligowski, Pawel Kiper

**Affiliations:** 1 Laboratory of Rehabilitation Technologies, IRCCS San Camillo Hospital, 30126 Venice, Italy; luisa.cacciante@hsancamillo.it; 2 Faculty of Health Sciences, Jan Dlugosz University in Czestochowa, 42-200 Czestochowa, Poland; 3 Department of Physical Education and Physiotherapy, Opole University of Technology, 45-758 Opole, Poland; s.rutkowski@po.edu.pl (S.R.); a.rutkowska@po.edu.pl (A.R.); katarzyna.kacperak@gmail.com (K.K.); 4 Department of Physiotherapy, University School of Physical Education in Wroclaw, 51-612 Wroclaw, Poland; tomasz.kuligowski@awf.wroc.pl; 5 Physical Medicine and Rehabilitation Unit, Azienda ULSS 3 Serenissima, 30126 Venice, Italy; pawel.kiper@aulss3.veneto.it

**Keywords:** telerehabilitation, speech-language treatment, telehealth, COVID-19, speech-language rehabilitation

## Abstract

The COVID-19 pandemic brought out the need to deliver health care services at a distance in the form of telerehabilitation (TR). This study aimed to analyse the Italian speech and language therapists’ (SLTs) opinions on the feasibility of the TR in the field of speech-language therapy during the COVID-19 pandemic. We developed an anonymous survey to determine the SLTs’ opinions on feasibility of TR during lockdown caused by COVID-19. We analysed the survey’s answers provided by 136 SLTs. Cronbach’s alpha coefficient showed good reliability of the survey. The SLTs working previously with TR showed better judgements regarding this method. The comparison analysis between TR and face-to-face treatment delivery showed statistically significant differences as follows: “importance” (4.35 vs. 3.32, *p* = 0.001), “feasibility” (3.37 vs. 2.11, *p* < 0.001), “alternative form” (3.64 vs. 2.58, *p* = 0.001) and “comparison” (2.24 vs. 1.69, *p* < 0.001), but not with “familiarity” (*p* = 0.81). The survey showed that most of the Italian SLTs were not satisfied with TR systems. SLTs who used TR previously had a better opinion on this treatment modality. Experience and familiarity with TR systems were key factors for the use of this new rehabilitation modality.

## 1. Introduction

Due to the pandemic caused by SARS-CoV2, health care services have required radical changes in the management of health care delivery and the safety of patients and hospital staff, worldwide. Indeed, since December 2019 the world has faced a new virus (SARS-CoV2) which, between February and April 2020, caused the first lockdown in many countries [1]. This situation has created several issues in the delivery of health care services. The problems caused by lockdown were related not only to the management of COVID-19 patients. All treatments, diagnostics and counselling delivery were significantly reduced. Some health care services were suspended, while others were limited only to emergencies. In this difficult period, it was necessary to find additional forms of communication and health delivery. Thus, to communicate with patients and to maintain treatment delivery, a very well-known technology was identified as a potential solution to overstep these issues. In-depth telehealth technology was already used in terms of counselling or treatment, encompassing telemedicine, telehealth care, telerehabilitation (TR) and many others [2]. Telehealth technology allows providing assessment, monitoring, prevention, intervention, supervision, education, consultation and counselling services by using videoconference in synchronous or asynchronous ways [3]. One of the clinical fields in which this technology was found to be useful is speech-language therapy. People with speech and language disorders in rural and remote areas may be at a disadvantage because of poor access to speech and language pathology services [4]. Hence, TR for speech and language disorders, which can be provided remotely for both adults and paediatric patients, could cover a wide range of treatments [5].

In the speech and language field, we can observe a growing body of evidence that underlines the validity and reliability of TR treatment delivery: several studies showed good validity of TR in both assessment and treatment of speech and language disorders [6,7] and in the assessment of speech and voice disorders in individuals with Parkinson’s Disease and hypokinetic dysarthria [8]. From the available evidence, it can be confirmed that TR applied to lexical deficits in chronic stroke patients is feasible and comparable to face-to-face treatment [9]. A study conducted by Ora and colleagues showed that TR for post-stroke aphasia is feasible and acceptable, with high satisfaction levels among patients and pathologists. However, authors concluded that access to clinical and technical expertise is needed when developing TR services [10]. Furthermore, Latimer et al. reported that TR for people with long-standing aphasia has the potential to reduce costs associated with health services management [11]. TR also had advantageous results in the treatment of fluency disorders: a series of research trials provided evidence that the use of TR technology is feasible, effective, and yields satisfactory clinical outcomes in the treatment of stuttering in children, adolescents, and adults [12,13,14]. Evidence showed that TR is a viable method to provide services for children with special needs and can be used to support the delivery of speech-language therapies in schools [5].

Despite the wide literature related to TR application, the pandemic situation made TR a highly discussed method for treatments delivery. For example, a study by Rettinger et al. evaluated Austrian therapists’ (i.e., physical therapists, speech-language therapists, and occupational therapists) attitudes towards teletherapy, including perceived barriers, during and before the COVID-19 lockdown. Authors found that therapists perceived the lack of professional training as a barrier to the implementation of teletherapy intervention, as well as a certain impersonality of care. Nearly half of the surveyed therapists reported that teletherapy is impersonal, and more than a half agreed that they urgently needed physical contact with their patients [15]. Indeed, clinicians’ perspectives and opinions could differ from the results of previous research studies, mainly due to technology-specific issues and resistance to change from therapists [16]. It is necessary therefore to analyze what is the perception on the feasibility of TR from clinicians’ points of view, so that the TR does not remain confined to scientific research alone, but could also be disseminated among clinicians. The integration between the results of previous research and the opinions of clinicians would allow a better implementation of the TR system, making it more effective for different types of pathologies.

Within this context, this study aimed to analyse the Italian speech and language therapists’ (SLTs) opinion on the feasibility of TR, as well as to study any differences that may exist on the opinion about TR between those who used TR during COVID-19 pandemic and those who continued to provide face-to-face treatments.

## 2. Materials and Methods

### 2.1. Design and Participants

The study was designed as a qualitative research study and involved the collection of data without experimental intervention. However, the procedures of data acquisition and storing were executed in accordance with the ethical standards on human experimentation and in accordance with the Declaration of Helsinki 1975, revised Hong Kong 1989. The study was designed and carried out according to the qualitative research guidelines (SRQR guidelines) [17]. We developed an anonymous survey for the SLTs, available online during the period between the 1st and the 20th of June 2020. The survey was developed ex novo by an SLT through Google Forms (i.e., an online and free survey administration app). The target group involved in this study were SLTs. The information from the survey was collected in Italy. Only professionally active SLTs were considered eligible with no restrictions to clinical experience and specialty.

### 2.2. Outcomes

The analysis of the study aims was based on the answers obtained from the survey. To better understand clinicians’ perceptions on the feasibility of TR, we divided the survey into five indicators, which together contributed to analyse the aim of this study. Thus, the five indicators were associated to five main questions, as follows:

1. Level of knowledge and familiarity with TR before COVID-19 pandemic, that we called “familiarity”.
2. Opinion about the importance of providing treatments through TR systems in the context of the country lockdown, that we called “importance”.
3. Opinion on the feasibility of TR treatments in different fields of speech-language rehabilitation, that we called “feasibility”.
4. Opinion on the use of TR as an alternative form of speech-language treatment. This was called “alternative form”.
5. SLTs’ judgements on the comparison between TR and face-to-face treatment, that was called “comparison”.

Participants were asked to provide an answer related to each question, based on a Likert scale (from one to five, where one represented “very low” and five represented “very high”) [18].

The survey comprised a first part for the collection of demographic data, and a second part including 10 questions. Five questions explored whether SLTs used TR during COVID-19 and referred to the presence or absence of issues that SLTs faced during the use of TR, or the reasons why they could not use this kind of treatment (for those who did not use TR during COVID-19). The remaining five questions were based on a Likert scale and explored the domains previously reported (i.e., “familiarity”, “importance”, “feasibility”, “alternative form”, “comparison”).

At the end of the survey, a space for comments was left, in order to collect qualitative information, opinions, comments, critical issues, arguments, ideas and suggestions from the SLTs.

### 2.3. Statistical Analysis

The reliability of the survey was analysed through Cronbach’s alpha. The results were collected in an Excel spreadsheet and subsequently subjected to statistical analysis using SPSS Statistics software (version 26, IBM, Armonk, NY, USA). As normality tests (Shapiro–Wilk test) revealed that none of the variables followed a normal distribution, non-parametric tests were used. The Mann–Whitney U test was used to compare the answers of participants who used TR as a method to deliver treatment during the COVID-19 pandemic and those who did not have any knowledge of TR. The level of significance was set at α < 0.05.

## 3. Results

### 3.1. Baseline Characteristics

Cronbach’s alpha coefficient showed good reliability of the survey (alpha reliability = 0.74; standardised alpha = 0.77) [19]. We analysed the survey’s answers provided by 136 SLTs (9 men and 127 women, which is consistent with existing literature on gender imbalance highlighted in other countries [20]). The demographic characteristics of the analysed group are shown in Table 1.

### 3.2. Between Group Comparison

**Figure 1:**
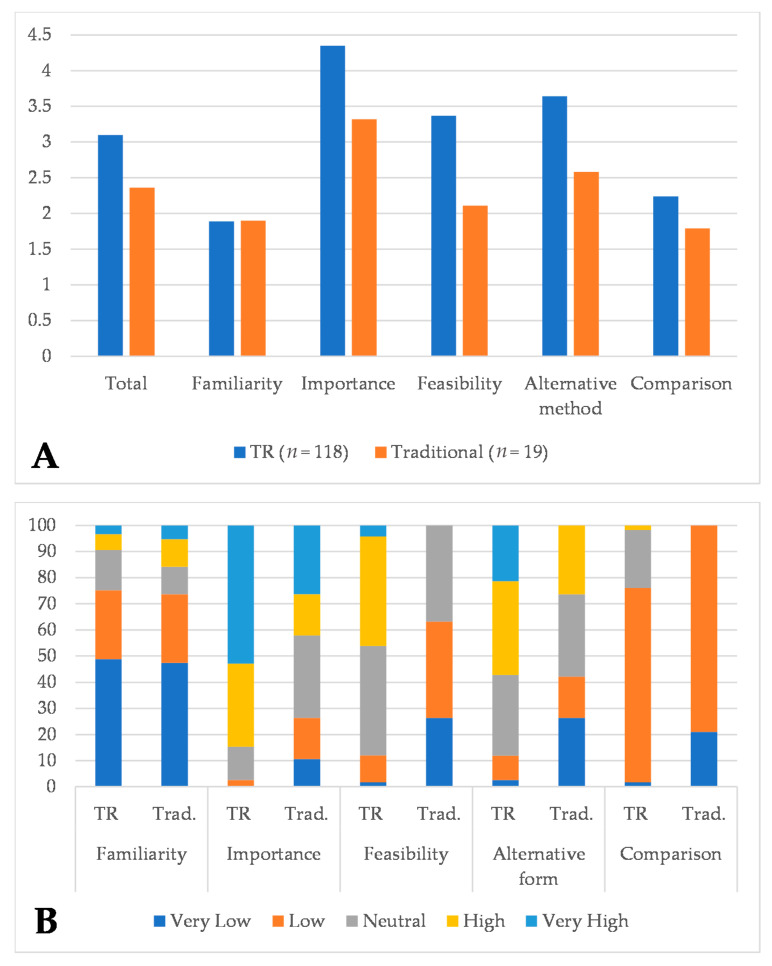
Domains comparison between telerehabilitation (TR) and traditional therapy in mean differences (**A**) and percentages results (**B**).

Table 2 illustrates the percentage distribution of individual responses (Table 2), and Table 3 shows the average results in each area, along with a comparison of the total score in the study groups (Table 3). Figure 1 displays domains comparison between TR and traditional therapy (Figure 1). SLTs who used TR as a method to deliver treatment during the COVID-19 pandemic indicated higher survey scores in all surveyed areas, except for “familiarity”, as compared to SLTs who did not use this method. There was a statistically significant difference between groups in overall survey score on a 5-point Likert scale (3.10 vs. 2.36, *p* < 0.001), also for outcomes related to “importance” (4.35 vs. 3.32, *p* = 0.001), “feasibility” (3.37 vs. 2.11, *p* < 0.001), “alternative form” (3.64 vs. 2.58, *p* = 0.001) and “comparison” (2.24 vs. 1.69, *p* < 0.001), but not to “familiarity” (*p* = 0.81).

## 4. Discussion

This study showed that the level of knowledge related to the TR was low before the COVID-19 pandemic, and that this situation introduced some changes in the way the Italian SLTs deliver treatments in their clinical practice. Indeed, most of the SLTs declared to be not familiar with the remote treatment that can be provided through this form of rehabilitation before the pandemic situation. Such a result could indicate that there is a lack of accessibility to innovative technologies probably due to the elevated costs of systems or even to the low possibility for a training of the SLTs. Another reason could be the fact that the need for telehealth was not given so much attention in the SLT field before the pandemic, except for specific geographical reasons. Indeed, because of the pandemic emergency, many clinicians have used TR to limit in-person contact, a necessary step to reduce the risk of exposure [21]. Despite the low level of familiarity with TR underlined by Italian SLTs before the COVID-19 pandemic, recent studies showed how the use of TR during the COVID-19 pandemic increased its acceptance by clinicians [22,23]. Negrini et al. demonstrated that the use of TR was feasible and acceptable during COVID-19 [23]. In another recent study, the authors observed that 85% of physical therapy sessions provided during the COVID-19 pandemic were administered using TR, and all physical therapists who participated in the study conducted at least one TR session, indicating a 100% adoption and an increased use of TR after the COVID-19 pandemic [22].

On the other hand, we observed that SLTs showed to have a very high interest in TR, by giving importance to treatments provided remotely. In relation to the opinion on feasibility, several issues were raised in the survey. The major issue was related to problems with patients’ compliance, given the lack of physical contact between therapists and their patients. This study showed that the impersonality of care seems to be the biggest barrier for the implementation of telehealth systems, similar to findings reported in a study by Rettinger et al. [15]. Another issue for the SLTs who delivered TR treatments during the lockdown was related to internet connection problems, which made treatments more difficult to provide, and negatively influenced the feasibility evaluation. This result is consistent with the results from Scott et al., who identified technology-specific issues as one of the top barriers for the implementation of telemedicine [16].

Based on SLTs’ answers, those who used TR during the pandemic showed more positive judgements about the consideration of TR as a good alternative form of speech-language therapy and as a valid alternative to conventional face-to-face rehabilitation, demonstrating that experience and familiarity are key factors for the use of this new rehabilitation modality. Although some SLTs took a neutral or high positive position, many others, especially those who have not used TR, disagreed with this alternative form of rehabilitation. This can be considered as a natural reaction, evoked due to the uncertainty about the outcomes and the clinical efficacy of TR [24]. Indeed, by analysing the differences between therapists who used TR during the pandemic and those who did not have contact with this method, a more positive attitude towards TR is visible in people who familiarised with TR. Interestingly, both groups declared a similar starting level of familiarity. However, the opinion of therapists using TR during the COVID-19 pandemic was significantly more positive towards TR. This may indicate “fear of the unknown” in people who have not used this method, or even to a “resistance to change”, as observed in previous literature [25,26,27]. Therefore, it seems extremely important to increase the accessibility of TR through various types of training or courses. Indeed, professional training was shown to have the potential to remove barriers that hinder telehealth implementation [28].

Finally, the opinion on the comparison between TR and conventional treatment showed that most of the SLTs considered it worse than conventional face-to-face treatment. The main problem arose from SLTs’ comments was related to the lack of relational aspects when delivering a treatment remotely. Most of the SLTs perceived the lack of contact, the lack of direct interaction with the patient, and the impossibility to perceive all the non-verbal communication aspects as barriers to the achievement of significant improvements. This can be due to the importance that SLTs give to the co-presence, which is seen as the factor that highly influences the therapeutic relationship and, consequently, may influence the efficacy of rehabilitation. This aspect is stressed in the field of the rehabilitation of children with autism spectrum disorders: especially in this case, with parents who have some difficulties in managing the situation, it would be fundamental to have direct contact with the patient, in order to create a greater involvement during the therapy session. Moreover, it seems that dysphagic patients are considered another category of patients that could not benefit from TR. In fact, based on SLTs’ opinions, it would be more difficult to train this kind of patient to the use of compensation manoeuvres or facilitating postures without having direct contact with them. A facilitator in this context could be a person proficient enough to use technology, who helps the patient while attending an online session, ensuring that therapy is smoothly conducted [24]. Moreover, TR can be used in conjunction with face-to-face treatment, exploiting the strengths of both modalities. Some SLTs noted that TR may not be excluded a priori, but it could be very useful in some cases, by selecting proper patients, establishing specific setting rules, and having appropriate devices.

It is worth noting that current evidence suggests the feasibility of TR and its well-acceptability by both therapists and patients [9,29]. Indeed, in literature, many experiences of TR for motor [30], cognitive [31], and speech-language rehabilitation [32] have been published. In the great majority of these studies, we can face the so-called “closed” systems, which are useful for research (i.e., enrolment of selected patients according to research protocol), but they seem to be unavailable for community health services, especially in the pandemic reality, where the research protocols cannot be applied. On the other hand, it is important to highlight that the equipment for teletherapy is expensive, as shown in previous studies on telehealth technologies [33,34,35]. However, it was demonstrated that the global costs of teletherapy were lower in comparison with those of the current rehabilitative home treatments [36]. Thus, the difference in the perception and opinion about the usefulness, feasibility, and efficacy of TR, between SLTs in Italy and worldwide, may be due to the lack of proper equipment in health care services. This was an observation underlined by several therapists in the comments part of the applied survey. Some of them reported that they cannot deliver treatments remotely during the lockdown because the rehabilitation centres where they work did not have available equipment to deliver TR treatments. Thus, there is the need to develop and share professional, technical standards and guidelines to ensure appropriate TR application, and to create new infrastructures to support TR also in the territorial health care services. These needs already existed [5], but now the current pandemic highlighted them in a more critical and evident way. This study provided new insights related to the feasibility and the importance of TR within the speech-language field from clinicians’ points of view, contributing to the future implementation of TR system according to therapists’ and, indirectly, to patients’ needs.

### Study Limitations

Some limitations need to be addressed. Firstly, in the included sample, a large discrepancy in the size of the studied groups can be noted. It results from the fact that during the pandemic, many active SLTs worked remotely, therefore it was difficult to find therapists who did not use TR. Secondly, the study used an original questionnaire, the results of which are difficult to relate directly to other studies. Finally, the questionnaire was developed by an SLT and not by a multidisciplinary team, which would include also participants from different fields. However, the study targeted SLTs, so we felt it necessary to develop the questionnaire by SLT. The above limitations should be considered when interpreting the results of this study.

## 5. Conclusions

This study aimed to analyse the feasibility of TR in the field of speech-language therapy during the COVID-19 pandemic and to observe how the lockdown caused by COVID-19 has led to changes in the rehabilitative methods used by SLTs. The survey we used showed that most of the Italian SLTs were not familiar with TR systems before the pandemic and that they are not satisfied with this kind of modality to provide treatments. There are several critical issues provided by SLTs, ranging from the lack of co-presence and relational aspects to problems related to the internet connection and the lack of specific equipment. All these issues negatively influenced the opinion of SLTs about the feasibility of TR. Following the survey’s answers, we cannot confirm that TR is feasible for Italian SLTs. It seems that the conventional face-to-face setting is much preferred over TR treatment. However, the opinion on TR changes with more exposure to this method. The actual challenge regards the lack of professional technical standards and guidelines to ensure appropriate application of speech-language TR not only in a research context, but also in a clinical one. Our results are consistent with the conclusions drawn by Regina Molini-Avejonas et al., that telehealth does pose an advantage over non-telehealth. However, certain barriers, such as technology-related issues and lack of training and acceptance, need to be resolved [37]. Further research is needed to develop new TR devices and systems suitable for different kinds of patients, usable for most therapists, sustainable for health services and feasible for all the SLTs and patients.

## Figures and Tables

**Table 1:** Demographic characteristics of the SLTs.

Characteristics	Participants Data	Respondents (Percentage)
Sex (*n*/%)	Men	9 (6.6%)
	Women	127 (93.4%)
Year (*n*/%)	22–30	69 (50.7%)
	31–39	28 (20.6%)
	40–48	22 (16.2%)
	49–57	13 (9.6%)
	58–66	4 (2.9%)
Field of intervention (*n*/%)	Adult and geriatric	19 (14%)
	Paedriatic	117 (86%)
Type of patients treated (*n*/%)	SLI	111 (81.6%)
	Learning disorders	101 (74.3%)
	ASD	75 (55.1%)
	Aphasia	33 (24.3%)
	Dysarthria	33 (24.3%)
	Dysphonia	32 (23.5%)
	Hearing loss	30 (22.1%)
	Dysphagia	29 (21.3%)
	Deafness	25 (18.4%)
	Apraxia	22 (16.2%)
	Intellectual disability	2 (1.5%)
	Others	1 (1%)

**Table 2:** Survey’s answers related to the TR, *n* (%).

Domains	Answers	Total (*n* = 136)	TR (*n* = 117)	Traditional (*n* = 19)	*p*-Value *
Familiarity	Very Low	66 (48.53)	57 (48.72)	9 (47.36)	0.91
	Low	36 (26.47)	31 (26.50)	5 (26.32)	0.98
	Neutral	20 (14.71)	18 (15.38)	2 (10.53)	0.58
	High	9 (6.62)	7 (5.98)	2 (10.53)	0.46
	Very High	5 (3.68)	4 (3.42)	1 (5.26)	0.69
Importance	Very Low	2 (1.47)	0 (0.00)	2 (10.53)	-
	Low	6 (4.41)	3 (2.56)	3 (15.79)	0.01
	Neutral	21 (15.44)	15 (12.82)	6 (31.57)	0.04
	High	40 (29.41)	37 (31.63)	3 (15.79)	0.16
	Very High	67 (49.26)	62 (52.99)	5 (26.32)	0.03
Feasibility	Very Low	7 (5.15)	2 (1.71)	5 (26.32)	<0.01
	Low	19 (13.97)	12 (10.26)	7 (36.84)	<0.01
	Neutral	56 (41.18)	49 (41.88)	7 (36.84)	0.68
	High	49 (36.03)	49 (41.88)	0 (0.00)	-
	Very High	5 (3.68)	5 (4.27)	0 (0.00)	-
Alternative form	Very Low	8 (5.88)	3 (2.56)	5 (26.32)	<0.01
	Low	14 (10.29)	11 (9.40)	3 (15.79)	0.40
	Neutral	42 (30.88)	36 (30.77)	6 (31.57)	0.94
	High	47 (34.56)	42 (35.90)	5 (26.32)	0.42
	Very High	25 (18.38)	25 (21.37)	0 (0.00)	-
Comparison	Much Worse	6 (4.41)	2 (1.71)	4 (21.05)	<0.01
	Worse	102 (75.00)	87 (74.36)	15 (78.95)	0.67
	Neutral	26 (19.12)	26 (22.22)	0 (0.00)	-
	Better	2 (1.47)	2 (1.71)	0 (0.00)	-
	Much Better	0 (0.00)	0 (0.00)	0 (0.00)	-

* According to Chi-square test; TR: refers to clinicians who used TR during COVID-19 pandemic; Traditional: refers to clinicians who did not use TR during COVID-19 pandemic.

**Table 3:** Comparison between TR and conventional face-to-face treatment.

Domains	Answers	TR (*n* = 117)	Traditional (*n* = 19)	*p*-Value *
Total	Mean (SD)	3.10 (0.60)	2.36 (0.65)	<0.001
	Median (IQR)	3.20 (2.6–3.6)	2.60 (2.0–2.8)	
Familiarity	Mean (SD)	1.89 (1.09)	1.90 (1.04)	0.81
	Median (IQR)	2 (1.0–2.5)	2 (1.0–3.0)	
Importance	Mean (SD)	4.35 (0.80)	3.32 (1.34)	0.001
	Median (IQR)	5 (4.0–5.0)	3 (2.0–5.0)	
Feasibility	Mean (SD)	3.37 (0.79)	2.11 (0.81)	<0.001
	Median (IQR)	3 (3.0–4.0)	2 (1.0–3.0)	
Alternative method	Mean (SD)	3.64 (1.00)	2.58 (1.17)	0.001
	Median (IQR)	4 (3.0–4.0)	3 (1.0–4.0)	
Comparison	Mean (SD)	2.24 (0.50)	1.79 (0.42)	<0.001
	Median (IQR)	2 (2.0–2.00)	2 (2.0–2.0)	

* According to Mann-Whitney U test; TR: refers to clinicians who have been using TR during COVID-19 pandemic; Traditional: refers to clinicians who have not been using TR during COVID-19 pandemic; IQR: Interquartile range.

## Data Availability

Data is available from the corresponding author on a reasonable request.
